# Molecular Characterization of Clinical Isolates of Methicillin-Resistant *Staphylococcus aureus* from Chonburi, Thailand

**DOI:** 10.3390/pathogens14050406

**Published:** 2025-04-24

**Authors:** Patcharawalai Wassanarungroj, Panida Nobthai, Sirigade Ruekit, Apichai Srijan, Prawet Sukhchat, Oralak Serichantalergs, John M. Crawford, Brett E. Swierczewski, Sidhartha Chaudhury, Paphavee Lertsethtakarn

**Affiliations:** 1 Department of Bacterial and Parasitic Diseases, Walter Reed Army Institute of Research-Armed Forces Research Institute of Medical Sciences (WRAIR-AFRIMS), 315/6 Rajvithi Rd., Bangkok 10400, Thailand; PatcharawalaiW@afrims.org (P.W.); PanidaN@afrims.org (P.N.); SirigadeR.fsn@afrims.org (S.R.); pap.srijan@gmail.com (A.S.); Sidhartha.Chaudhury.mil@afrims.org (S.C.); 2 Queen Sirikit Naval Hospital, Chonburi 20180, Thailand; 3 US Army Research Institute of Chemical Defense, Gunpowder, MA 21010, USA; john.m.crawford58.mil@health.mil; 4 US Army Medical Research Institute of Infectious Diseases, Frederick, MA 21702, USA; brett.e.swierczewski.mil@health.mil

**Keywords:** MRSA, SCC*mec*, MLST, PFGE, *spa*, PVL, Thailand

## Abstract

*Staphylococcus aureus* (*S. aureus*), especially methicillin-resistant *S. aureus* (MRSA), remains a major public health concern. This study reports the antimicrobial resistance profiles and molecular characteristics of 31 *S. aureus* isolated during 2017–2018 from inpatient and outpatient clinical specimens from Queen Sirikit Naval Hospital (QSH) in Chonburi province, Thailand. All isolates were tested for antimicrobial susceptibility. Staphylococcal cassette chromosome *mec* (SCC*mec*) typing, Panton–Valentine leukocidin (*pvl*) toxin, pulsed-field gel electrophoresis (PFGE), multilocus sequence typing (MLST), and staphylococcal protein A (*spa*) typing were performed. Twenty-seven isolates were confirmed to be MRSA and exhibited resistance to up to seven antibiotics classes. The main MLST type was SCC*mec* type II (51.9%) and ST764 (55.6%). Five *spa* types were identified with t045 (55.6%) as the major type. All 31 *S. aureus* isolates were grouped into seven types using PFGE with the SCC*mec*II-ST764-t045 clone being the most prevalent. Overall, our findings reveal that the *S. aureus* isolates in this study differ from previous reports in Thailand, indicating a potential shift in local strains, highlighting the need for ongoing molecular surveillance of multidrug resistance patterns of MRSA in Southeast Asia.

## 1. Introduction

*Staphylococcus aureus* (*S. aureus*), a Gram-positive bacterium, is a significant pathogen responsible for a wide range of infections, both in community settings and healthcare facilities. *S. aureus* can cause conditions ranging from mild infections like folliculitis to severe, life-threatening diseases such as endocarditis and osteomyelitis [1,2]. Among these, methicillin-resistant *S. aureus* (MRSA) is one of the most common hospital- and community-acquired pathogens associated with morbidity and mortality. The Centers for Disease Control and Prevention (CDC) reported that MRSA infections led to an estimated 20,000 deaths in the US in 2017 [3]. On a global scale, MRSA was attributed to 100,000 deaths worldwide in 2019 [4]. In Thailand, estimates for the prevalence of MRSA varies widely from as low as 10–20% [5,6,7] to as high as 40–60% [8,9], with the highest prevalence typically observed in healthcare settings [7] and in tertiary hospitals [6].

Methicillin was first introduced in 1959 as a treatment for staphylococcal infections. The initial case of MRSA was reported in London within two years of its introduction [10]. Over the past few years, the effectiveness of treatments for MRSA infections has significantly diminished due to increased resistance to various classes of antibiotics [1]. Generally, MRSA is divided into two main groups: hospital-associated (HA) and community-associated (CA) MRSA [11]. In clinical settings, the infections caused by HA-MRSA strains are associated with high mortality and morbidity. These strains are usually multidrug resistant (MDR), defined as resistance to at least one agent in three or more antimicrobial classes, which limits the number of antibiotics available to effectively treat the infection [12]. In addition to the risks of HA-MRSA, a growing population of CA-MRSA strains have been reported to express virulence factors, such as Panton–Valentine leucocidin (PVL).

The characterization of methicillin-resistant *S. aureus* (MRSA) is primarily based on Staphylococcal cassette chromosome *mec* (SCC*mec*) types, a mobile genetic element that is the defining feature of MRSA, which is classified into major types I to XIII, along with various subtypes [1]. Multiplex PCR assays have been developed to identify all major SCC*mec* types [13,14]. Other methods, such as pulsed-field gel electrophoresis (PFGE) [15], multilocus sequence typing (MLST) [16] and whole genome sequencing (WGS) [17,18] have also been employed to characterize *S. aureus*. In addition to molecular typing using PFGE, MLST, and WGS, there is an additional typing provided by an analysis of the Protein A gene (*spa*), a virulence factor found on the surface of *S. aureus* that prevents the bacteria from phagocytosis by the host immune system. A hypervariable region, Xr, in the *spa* gene is conserved among *S. aureus* strains and has been used for the development of a single-locus sequence typing technique for *S. aureus* [19].

In addition to molecular typing, characterization of virulence factors can yield additional insight into the epidemiology and pathogenesis of MRSA. Panton–Valentine leucocidin (PVL), a cytotoxic toxin encoded by *lukS-PV* and *lukF-PV*, is well known for its induction of apoptosis in human polymorphic leukocytes causing cell lysis as well an association with severe pneumonia and skin/soft tissue infections caused by *S. aureus* [20,21]. PVL mainly targets and destroys polymorphonuclear leukocytes, monocytes, and macrophages, triggering cell death and promoting the release of pro-inflammatory cytokines and nuclear factor-kappa B. PVL production results in necrotizing damage such as lung injury and deep-seated skin and soft tissue infections [20]. *S. aureus* strains producing PVL are reported to be more virulent, highly transmissible, and more toxic, with a stronger correlation to increased mortality and morbidity compared to strains lacking PVL [22].

Previously, we conducted a general AMR bacteria surveillance study using inpatient and outpatient samples collected from Queen Sirikit Naval Hospital in Chonburi, Thailand from 2017 to 2018 that identified 431 ESKAPEE isolates, including 25 *S. aureus* isolates [23]. Here, we augment that sample set with additional *S. aureus* isolates collected from the same site and carry out an in depth molecular characterization of *S. aureus* isolates including identification of SCC*mec* types; molecular typing with PFGE; analysis of *spa*, *mecA*, and *pvl* genes; and whole genome sequencing. Our findings provide a comprehensive molecular profile of clinical *S. aureus* isolates in this region and their association with antibiotic drug resistance.

## 2. Materials and Methods

### 2.1. Bacterial Strains

*S. aureus* was isolated and identified as part of a surveillance study of multidrug resistant ‘ESKAPEE’ pathogens, which includes *Enterococcus faecium*, *Staphylococcus aureus*, *Klebsiella pneumoniae*, *Acinetobacter baumannii*, *Pseudomonas aeruginosa*, *Enterobacter* spp., and *Escherichia coli*, at the microbiology laboratory at Queen Sirikit Naval Hospital, Chonburi, Thailand from April 2017 to May 2018 [23]. Thirty-one MDR *S. aureus* isolates were transferred to Walter Reed Army Institute of Research—Armed Forces Research Institute of Medical Sciences (WRAIR-AFRIMS) in Bangkok, Thailand for further molecular investigations. Isolates were grown on sheep blood agar plates and trypticase soy agar plates followed by an incubation under aerobic conditions at 35 ± 2 °C for 18–24 h in preparation for downstream molecular analysis.

### 2.2. S. aureus Phenotypic Characterization and Antimicrobial Susceptibility Testing

Biochemical identification and antimicrobial susceptibility testing were performed with the BD Phoenix M50 using the PMIC/ID 55 panel, according to the manufacturer’s instructions (BD Diagnostics, Sparks, MD, USA). Results interpretation adhered to the Clinical and Laboratory Standards Institute (CLSI) guidelines reported in 2017 [24]. *Staphylococcus aureus* ATCC 29213 and *Enterococcus faecalis* ATCC 29212 were used for quality controls. The antimicrobial susceptibility tests for *S. aureus* isolates using the PMIC/ID included amikacin, ampicillin, ciprofloxacin, clindamycin, erythromycin, gentamicin, linezolid, high level mupirocin, oxacillin, penicillin, rifampin, teicoplanin, tetracycline, trimethoprim/sulfamethoxazole, and vancomycin. MRSA screening was conducted based on the minimum inhibitory concentrations (MICs) of oxacillin and cefoxitin, with isolates exhibiting an oxacillin MIC ≥ 4 µg/mL and cefoxitin ≥ 8 µg/mL being classified as methicillin-resistant *S. aureus* (MRSA).

### 2.3. Genomic DNA Purification of S. aureus Isolates

Genomic DNA was extracted using DNeasy Blood & Tissue Kit (Qiagen GmbH, Hilden, Germany) according to the manufacturer instruction with a pretreatment lysis of Gram-positive bacterial cell walls. DNA concentration and quality assessment were determined using a NanoDrop spectrophotometer (Thermo scientific, Waltham, MA, USA). The extracted DNA was stored at −20 °C until analysis.

### 2.4. Real-Time PCR Detection of the mecA Gene

Real-time PCR was performed on all *S. aureus* isolates to detect the *mecA* gene, which is responsible for methicillin resistance. The real-time PCR assays were performed as previously described [25] on a CFX96 Touch Deep Well™ Real-Time PCR Detection System (Bio-Rad, Hercules, CA, USA) using the SensiFAST Probe No-ROX Mix (Bioline, London, UK). Real-time PCR results were analyzed using Bio-Rad CFX Manager software version 3.1 (Bio-Rad, Hercules, CA, USA). The threshold cycle (Ct) was set at 1000 relative fluorescence units (RFUs). *S. aureus* ATCC BAA-1768, *S. aureus* ATCC 25923, and sterile water were used as the positive control, negative control, and blank, respectively.

### 2.5. PCR Detection of Panton–Valentine Leukocidin (pvl) Toxin

All *S. aureus* isolates were tested for the presence of *pvl* genes (433 bp) with PCR as previously described [2] using a Mastercycler^®^ Nexus GX2 (Eppendorf, Framingham, MA, USA). The PCR reaction was composed of 25 μL containing 2 µL of DNA template, 1x Ampli*Taq* PCR buffer, 1.5 mM MgCl_2_, 100 µM of each dNTP, 0.4 µM of each primer (pvl-F and pvl-R), and 1.25 U of Ampli*Taq* DNA polymerase (Thermo Fisher Scientific, Waltham, MA, USA). The thermal cycling conditions were as follows: denaturation at 94 °C for 5 min; followed by 28 cycles of 94 °C for 1 min, 55 °C for 1 min, and 72 °C for 1 min; with a final extension at 72 °C for 10 min.

### 2.6. Identification of SCCmec Types

SCC*mec* typing was performed as outlined by Boye *et al*., 2007 for SCC*mec* type I to V [14]. The PCR amplification of SCC*mec* genes was carried out in a 25 µL reaction mix containing 2 µL of DNA template, 1x Ampli*Taq* PCR buffer, 1.5 mM MgCl_2_, 100 µM of each dNTP, and 1.25 U of Ampli*Taq* DNA polymerase (Thermo Fisher Scientific, Waltham, MA, USA). Primer concentrations were as follows: primers β and α3, 0.2 µM each; ccrC-F and ccrC-R, 0.25 µM each; 1272F1 and 1272R1, 0.08 µM each; and 5RmecA and 5R431, 0.1 µM each. The reaction was carried out on a Mastercycler^®^ Nexus GX2 (Eppendorf, Framingham, MA, USA). The thermal cycling conditions comprised denaturation at 94 °C for 5 min; followed by 28 cycles of 94 °C for 1 min, 55 °C for 1 min, and 72 °C for 1 min; with a final extension of 72 °C for 10 min. The SCC*mec* types were determined by comparing the banding patterns of the MRSA isolates with reference strains: ATCC BAA-44 (SCC*mec* type I), ATCC BAA-41 (SCC*mec* type II), NR-45889_BEI Resources (SCC*mec* type III), ATCC BAA-1768 (SCC*mec* type IV), and ATCC BAA-2094 (SCC*mec* type V).

Multiplex PCR assays for SCC*mec* type VI to XII were performed using two sets of primers that target SCC*mec* type VI to VIII and SCC*mec* type IX to XII [13]. The PCR reaction contained 25 μL with 2 µL of DNA template, 1x Ampli*Taq* PCR buffer, 1.5 mM MgCl_2_, 200 µM each dNTP, and 1.25 U of Ampli*Taq* DNA polymerase (Thermo Fisher Scientific, Waltham, MA, USA). For SCC*mec* type VI to VIII, 0.1 µM of each primer was used except 0.2 µM of Type VII. For SCC*mec* type IX to XII, 0.1 µM of each primer was used except 0.2 µM of Type IX and Type X. The thermal cycling condition was as follows: an initial denaturation at 94 °C for 5 min followed by 30 cycles of 94 °C for 1 min, gradient annealing at 50–60 °C for 1 min, and 72 °C for 1 min with a final extension at 72 °C for 10 min. All of the amplified products were sent for sequencing for confirmation (First BASE, Seri Kembangan, Malaysia).

An analysis of whole genome sequencing (WGS) data was performed on a strain that was not typeable using multiplex PCR assays described above. WGS was performed on Illumina MiSeq Benchtop sequencer using MiSeq Reagent Kit v3. The library was prepared using KAPA HyperPlus Library Preparation Kit and quantified using the KAPA Library Quantification Kit (Roche Diagnostics Corporation, Indianapolis, IN, USA) as described previously [23]. The sequences are available at the NCBI Sequence Read Archive (https://www.ncbi.nlm.nih.gov/sra, accessed on 13 January 2025) under BioProject no. PRJNA814829. Sequences of the untypeable isolates were edited and assembled using BioNumerics software version 7.6 (Applied Maths, Ghent, Belgium). The edited sequences were queried in SCC*mec*Finder, a web-based tool for Typing of Staphylococcal Cassette Chromosome *mec* in *Staphylococcus aureus* (https://cge.food.dtu.dk/services/SCCmecFinder/, accessed on 26 March 2025).

### 2.7. Polymorphism of the X Region Encoding S. aureus Protein A (spa Typing)

The PCR assay was performed as previously described [19] in a final volume of 50 μL containing 4 µL of DNA template, 1x Ampli*Taq* PCR buffer, 1.5 mM MgCl_2_, 100 µM of each dNTP, 0.1 µM each of spa-1113 F and spa-1514 R primer, and 1.25 U of Ampli*Taq* DNA polymerase (ThermoFisher Scientific, Waltham, MA, USA) on a Mastercycler^®^ Nexus GX2 (Eppendorf, Framingham, MA, USA) following Ridom SpaServer recommendations (https://spaserver.ridom.de/, accessed on 9 March 2023). Amplified products were purified and commercially sequenced (First BASE, Seri Kembangan, Malaysia). Spa types were assigned based on the repeat profile using BioNumerics software version 7.6 (Applied Maths, Ghent, Belgium), referencing the Ridom SpaServer database (http://www.spaserver.ridom.de, accessed on 3 May 2023).

### 2.8. Multilocus Sequence Typing (MLST) and Clonal Complex (CC)

MLST analysis was performed as described previously [16]. Seven housekeeping genes were amplified using PCR, each approximately 500 base pairs in length: carbamate kinase (*arcC*), shikimate dehydrogenase (*aroE*), glycerol kinase (*glp*), guanylate kinase (*gmk*), phosphate acetyltransferase (*pta*), triosephosphate isomerase (*tpi*), and acetyl coenzyme A acetyltransferase (*yqiL*). PCR reactions were performed using Ampli*Taq* Gold Master mixtures (Thermo Fisher Scientific, Waltham, MA, USA). The generated sequences were submitted to the *S. aureus* PubMLST database (http://pubmlst.org/saureus, accessed on 25 March 2025) to define the allelic profile using the alleles of the seven loci, which corresponded to a sequence type (ST) and to define a clonal complex (CC) based on ST groups that shared at least five of the seven alleles. New alleles and STs were deposited into the database. The distribution of CCs among the isolates was visualized using minimum spanning trees generated in BioNumerics software V.7.6 (Applied Maths, Ghent, Belgium).

### 2.9. Visualization of PCR Products and Analysis of Sequenced PCR Products

PCR products (6–8 µL) were visualized by electrophoresis on 1.5–1.8% *w*/*v* agarose gels with ethidium bromide (0.5 µg/mL) staining and the images were captured using a gel documentation system (Syngene, Cambridge, UK). PCR products were purified for sequencing using the Wizard SV Gel and PCR clean up system (Promega, Madison, WI, USA) and sent for sequencing to First BASE, Seri Kembangan, Malaysia. Sequences for MLST and *spa* typing were edited and analyzed using Bionumerics Version 7.6 software (Applied Maths, Ghent, Belgium).

### 2.10. Pulsed-Field Gel Electrophoresis (PFGE)

PFGE was performed on all of the *S. aureus* using a method previously described [15] with minor modifications. Briefly, a single colony of the *S. aureus* isolate was inoculated into 5 mL of brain heart infusion broth and incubated with shaking at 37 °C for 18–24 h. Genomic plugs were prepared, and the DNA was digested using 40 U *sma*I (Roche Diagnostics, Indianapolis, IN, USA). The digested plugs were loaded into the wells of a 1.8% agarose gel and run in 0.5x TBE using a CHEF Mapper system (Bio-Rad, Hercules, CA, USA) with the following parameters: 6 V, temperature 14 °C, initial switch time 5 s, final switch time 40 s, included angle 120, and a run time of 21 h. The *Salmonella* Braenderup H9812 strain (ATCC BAA664) digested with *Xba*I was included as a control marker. After electrophoresis, gels were stained with ethidium bromide solution (0.5 µg/mL), and DNA band patterns were visualized using a gel documentation system (Syngene, Cambridge, UK). Gel images were saved as TIFF files and analyzed using the BioNumerics software version 7.6 (Applied Maths, Ghent, Belgium). The phylogenetic tree was constructed to assess the relationship among *S. aureus* isolates with cluster analysis using the Dice coefficient and the unweighted pair group method (UPGMA). A dendrogram of isolates with over 80% similarity were clustered as having the same pattern.

## 3. Results

A total of 31 MDR *S. aureus* isolates were received and confirmed, accounting for 4.6% of the 670 ESKAPEE isolates received during the reporting period. A subset of these isolates (25) was included in the overall surveillance report [23]. Phenotypic and genotypic characterization identified 27/31 (87%) isolates to be MRSA based on MIC results and the presence of *mecA* using real time PCR, and the remaining 4/31 (13%) as MDR *S. aureus* isolates that were methicillin-susceptible *S. aureus* (MSSA). The distribution of MRSA according to specimen types were as follows: sputum 13/27 (48.1%), pus 6/27 (22.2%), blood 2/27 (7.4%), urine 2/27 (7.4%), wound exudate 2/27 (7.4%), central line 1/27 (3.7%), and radivac drain 1/27 (3.7%).

MRSA isolates in this study exhibited drug resistance against non-β-lactam antibiotics at varying degrees (Table 1). All isolates were resistant to ampicillin and penicillin. In addition, MRSA isolates were resistant to clindamycin (88.9%), erythromycin (88.9%), ciprofloxacin (85.2%), gentamicin (59.3%), tetracycline (55.6%), trimethoprim/sulfamethoxazole (11.1%), rifampin (7.4%), and mupirocin (3.7%) (Table 1). All but one of the 27 MRSA isolates were multidrug resistant (MDR), with resistance up to seven antibiotic classes, and one MSSA isolate was MDR with resistance to clindamycin and erythromycin.

SCC*mec* types I, II, IV, and V were identified among 26 MRSA isolates with one isolate that was not typeable. SCC*mec* type II was the most common 14/27 (51.9%), followed by SCC*mec* type I 5/27 (18.5%), SCC*mec* type V 4/27 (14.8%), and SCC*mec* type IV 3/27 (11.1%). The gel band patterns of SCC*mec* types I-V are shown in Supplemental Figure S1. The untypeable strain was further characterized for SCC*mec* type VI to XII but it remained untypeable. Further characterization with SCCmecFinder using WGS data (BioProject no. PRJNA814829, https://www.ncbi.nlm.nih.gov/sra/SRX15160234%5baccn, accessed on 13 January 2025) [23] did not yield any typing data. The isolate contained *mecA,* but a key SCC*mec* element was not detected, namely, *ccrA2* and *ccrB2*. It also contained the key type II genes (GenBank no. CP000736) *mec*I and *mec*R1 with 100% identity but only had a 51.09% sequence coverage to SCC*mec* type II. This isolate may represent a new or a variant of an existing SCC*mec* types where further characterization is needed.

**Figure 1:**
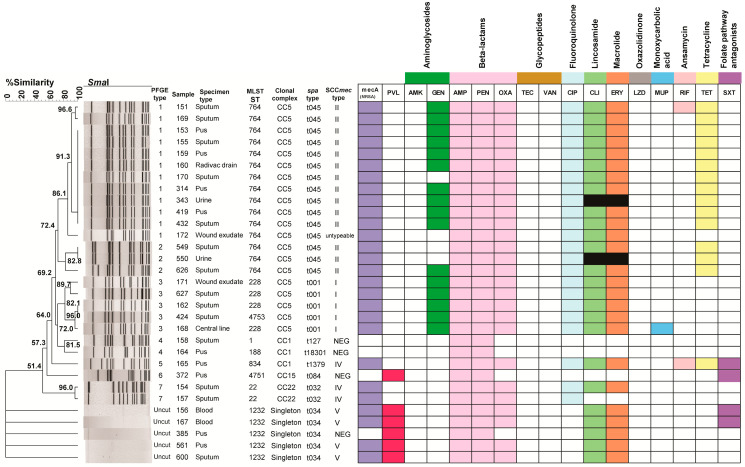
A dendrogram of PFGE clusters and genotypic relationships of reported *S. aureus* isolates. *Sma*I patterns were analyzed using the Dice coefficient method with the unweighted pair group method with averages (UPGMA) at 1.5% tolerance and 1.5% optimization settings. PFGE groups determined using cluster analysis are numbered from 1 to 7. The MLST ST types, clonal complex types, *spa* types, SCC*mec* types, *mec*A, PVL, and antibiotic resistance profiles are also included. Abbreviations for the antibacterial agents: amikacin (AMK), ampicillin (AMP), ciprofloxacin (CIP), clindamycin (CLI), erythromycin (ERY), gentamicin (GEN), linezolid (LZD), mupirocin (MUP), oxacillin (OXA), penicillin (PEN), rifampin (RIF), teicoplanin (TEC), tetracycline (TET), trimethoprim/sulfamethoxazole (SXT), vancomycin (VAN). Colored squares indicate resistance to listed antibiotics, white squares indicate sensitivity to listed antibiotics, and black squares indicate uninterpretable results from BD Phoenix M50 using the PMIC/ID 55 panel. Singleton refers to unique strains that do not belong to a larger clonal complex.

In this study, the *pvl* genes were present in 4/27 (14.8%) and 2/4 (50%) of MRSA and MSSA isolates, respectively. *pvl* genes have been reported to be associated with CA-MRSA strains harboring SCC*mec* type IV, V, VI, VII, and VIII [11]. In this study, all four PVL-positive MRSA isolates harbored SCC*mec* type V, while 3 isolates harboring the SCC*mec* type IV were PVL negative (Table 1 and Figure 1). Single-locus sequence typing of the *spa* gene revealed five different *spa* types among the 27 MRSA isolates with *spa* type t045 (55.6%) as the most common type, followed by t001 (18.5%), t034 (14.8%), t032 (7.4%), and t1379 (3.7%). The four MSSA isolates contained different *spa* types (t127, t084, and t034) and a new *spa* type, t18301, that was identified and submitted to https://www.spaserver.ridom.de/ (accessed on 9 March 2023) with GenBank accession no. MW118058 (Figure 1). This is the first report of the t18301 *spa* type.

To further characterize MRSA and to investigate the clonal relatedness of MRSA in this study, MLST was performed using an analysis of allelic sequences of short DNA fragments of seven *S. aureus* housekeeping genes [16]. All 27 MRSA isolates were classified into six different STs (Figure 1). ST764 was the most common (55.6%), followed by ST1232 and ST228 (both 14.8%), ST22 (7.4%), and ST834 (3.7%). New ST types ST4751 (https://pubmlst.org/bigsdb?page=profileInfo&db=pubmlst_saureus_seqdef&scheme_id=1&profile_id=4751, accessed on 26 March 2025) and ST4753 (https://pubmlst.org/bigsdb?page=profileInfo&db=pubmlst_saureus_seqdef&scheme_id=1&profile_id=4753, accessed on 26 March 2025) were identified in two isolates (BioProject no. PRJNA814829) [23]. ST4751 was the result of a novel combination of previously assigned allele sequences, indicating a recombination event. In contrast, ST4753 arose from new allele sequences, containing a single nucleotide change compared to a known allele, suggesting a point mutation or a recombination event. The four MSSA strains were identified as belonging to ST1, ST188, ST4751, and ST1232.

**Figure 2:**
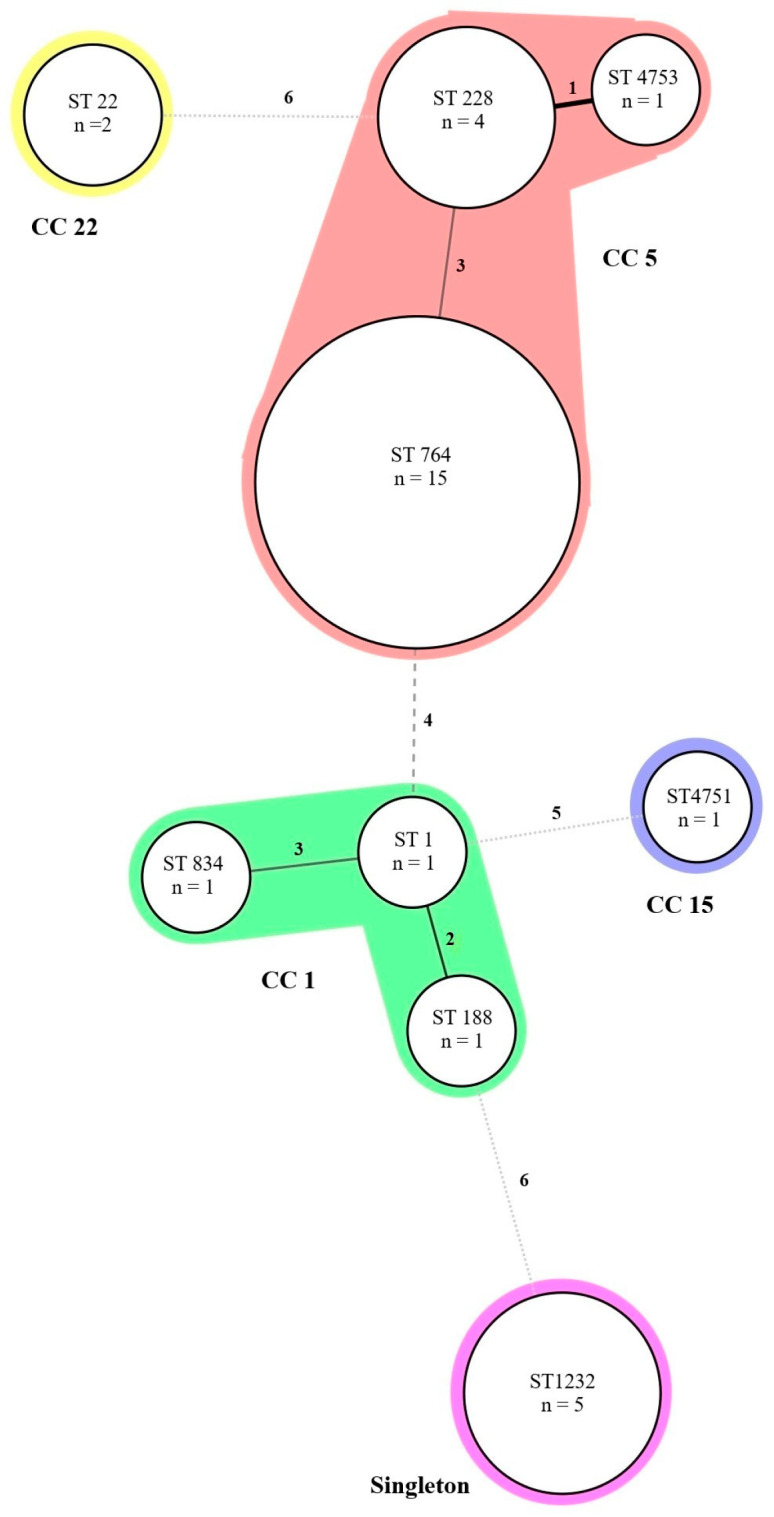
A minimum spanning tree (MST) illustrates the genetic relationships among 31 *S. aureus* (MRSA and MSSA) isolates based on multilocus sequence typing (MLST). Each node represents a distinct sequence type (ST). STs are assigned to clonal complexes (CCs) automatically using a script that runs against the PubMLST database. Lines connecting the nodes indicate genetic similarity, where shorter distances correspond to closer evolutionary relationships between STs. n = the number of isolates.

To further characterize MRSA and to investigate the clonal relatedness of MRSA in this study, MLST, PFGE and clonal complex analysis were performed. All 27 MRSA isolates were classified into six different STs (Figure 1). ST764 was the most common (55.6%), followed by ST1232 and ST228 (both 14.8%), ST22 (7.4%), and ST834 (3.7%). A dendrogram of percent similarity, calculated with Dice coefficients from the PFGE data using a cutoff of 80%, revealed seven major clusters of isolates designated as PFGE types 1 to 7 (Figure 1). The combined analysis of the PFGE pattern, MLST, *spa* type, SCC*mec* type, *mec*A gene, *pvl* gene, and drug resistance pattern are shown in Figure 1. Pattern 1 was the main PFGE cluster in this study (12/31, 38.7%, ST764-SCC*mec* II-t045), followed by pattern 3 (5/31, 16%, ST228-SCC*mec* I-t001 and ST4753-SCC*mec* I-t001) (Figure 1). Among these *S. aureus* isolates, five clonal complexes (CCs) were identified from MLST data and represented as a minimum spanning tree in Figure 2. CC5 is the major CC containing the most common ST764, ST228, and a new ST4753, and the SSC*mec* untypeable strain is included in this CC (Figure 2). Singletons included all of the SCC*mec* type V isolates identified in the study with one MSSA isolate. The remaining MSSA isolates were classified as CC1 and CC15. CC15 contains one of the new ST types, ST4751, as reported above.

## 4. Discussion

We carried out antimicrobial susceptibility testing and molecular typing on 31 *S. aureus* isolates collected from inpatient and outpatient samples at Queen Sirikit Hospital in Chonburi, Thailand. Overall, 27 isolates were classified as MRSA (*mec*A positive), 12 of which were resistant to five or more antimicrobial classes indicating a high level of drug resistance; four isolates were classified as MSSA (*mec*A negative), two of which were sensitive to all drug classes tested except β-Lactams. All isolates were susceptible to amikacin, linezolid, teicoplanin, and vancomycin, supporting the continued use of these drugs as treatment options for MRSA infection. The *S. aureus* isolates trend in Thailand, however, shows a significant increase from 2015 to 2019, stabilizing at a relatively high level until 2021 with a notable decline in 2022, followed by a partial rebound in 2023 [26].

SCC*mec* typing revealed that a majority of the MRSA isolates were SCC*mec* type II (51.8%). SCC*mec* type II carries various drug resistance genes, an integrated copy of staphylococcal plasmid pUB110 in the J3 region, and the Tn554 transposon in the J2 region conferring resistance to aminoglycoside, macrolide, lincosamide, and streptogramin (MLSB) [27,28]. Thus, it was not surprising that all SCC*mec* type II isolates were MDR. However, the prevalence of SCC*mec* type II in this study differs from other previous reports where SCC*mec* type III was the most commonly identified SCC*mec* type among MRSA isolates from various regions of Thailand [29,30,31]. The difference in this observation may be due to geographic and temporal differences that could suggest an epidemiological shift of the circulating SCC*mec* types in Thailand [31]. Our findings most closely reflect recent studies in China that reported a higher prevalence of SCC*mec* type II [32,33], compared those from India which showed a mixture of SCC*mec* type III and IV [34], and Japan where there has been a recent shift from SCC*mec* Type II to SCC*mec* Type IV [35,36].

SCC*mec* type I, a HA-MRSA prevalent in several countries (56.9%), was the second most prevalent type in our study, representing 18.5% of MRSA isolates [37]. Seven strains were SCC*mec* types IV and V, which are reported to be common in community-associated MRSA (CA-MRSA). Unlike SCC*mec* types I, II, and III, SCC*mec* type IV and V do not have any identifiable antibiotic resistance genes other than *mecA*, which has been used to explain its ability to achieve greater fitness and outcompete HA-MRSA strains that carry a larger number of genes on their mobile genetic element [38]. The prevalence of the SCC*mec* type differs geographically with reports including type I in southern Germany and Belgium; type II in England; type III in India, Iran and China; and type IV in Lebanon. This provides a basis for continued monitoring of SCC*mec* types among MRSA isolates [10,39,40].

The PVL-positive rate of MRSA strains in this study was low, similar to a report from Japan [11] in 2019. However, this is in contrast to a higher prevalence of PVL (>48%) reported in Colombia, India, and Saudi Arabia [11], indicating that PVL prevalence may be dependent on geography. When comparing PVL-positive versus PVL-negative MSSA isolates, we found that resistance to clindamycin, erythromycin, and trimethoprim/sulfamethoxazole was associated with PVL-positive MSSA isolates, indicating the potential for increased virulence. There was no observable association between the presence of PVL and drug resistance patterns among MRSA isolates except for the observation that two out of the four MRSA SCC*mec* type V isolates in our studies showed trimethoprim/sulfamethoxazole resistance, which was otherwise rare in this study. The combined virulence of PVL and MDR MRSA/MSSA could prove to be a challenge for effective therapeutic treatment.

We identified five different *spa* types among the 27 MRSA isolates with *spa* type t045 as the most common type, similar to a previous report from a hospital in western Thailand in 2021 [20], followed by t001, t034, t032, and t1379. The four MSSA isolates contained *spa* types t127, t084, and t034 and a newly identified *spa* type, t18301.

Molecular typing based on PFGE typing, MLST typing, *spa* typing, and clonal complex assignment were used to successfully classify *S. aureus* isolates into seven PFGE types, of which ST764-SCC*mec* II-t045 (PFGE type 1) was the most prevalent (12/26 or 46%), and five clonal complexes with CC5 as the most prevalent (20/31 or 65%). ST764 was recently recognized as a hybrid of the ST5 lineage of HA-MRSA with few virulence traits of CA-MRSA in Japan causing invasive infections and being carried by medical students [41]. ST764 SCC*mec* type II, with similar genetic characteristics to ST764-MRSA-II in Japan, was recently reported to be associated with severe hospital-acquired invasive diseases in Thailand as well [17], suggesting that it may have spread from Japan through healthy carriers [17,41]. Such spread was evident in reports noting that ST764-MRSA-II was also recently reported in Shanghai and Eastern China [33,42,43]. In contrast to previous reports that HA-MRSA strains ST239 SCC*mec* III and ST5-SCC*mec*II were the most common types in Thailand, this study reports ST764-SCC*mec* II-t045 to be common [30,31,37,44,45]. This is similar to Kondo et al., 2022 [17], in which this strain is highly similar to the recently emerged strain in Japan, indicating the intercontinental spread of this strain. Of the five isolates that could not be typed using PFGE, all were of the t034 *spa* type, which is consistent with other reports of untypeable *S. aureus* isolates [46,47]. The untypeability could be due to DNA methylation at the *Sma*I restriction site, CCCGGG, preventing it from being digested [48,49]. This hypervariable region of the *spa* gene and the epigenetic nature of this region may be a contributing factor to *S. aureus* virulence, specifically its ability to evade the host immune system [50].

Clonal complex assignment also provides additional information on the relatedness of these isolates where the grouping aligns similarly to the derived dendrogram from the PFGE data. CC5 was the major CC identified, aligning with reports that it is a significant global clonal complex that is associated with hospital-acquired MRSA [51]. The presence of ST1, a well-documented lineage in both hospital and community settings, in CC1, commonly associated with community MRSA strains, suggests a potential transmission between hospital and environmental settings [35]. This shift was demonstrated in northern Japan in bloodstream infections, where CC5 (ST5/ST764)-SCC*mec*II, which was predominant in 2017–2021, was surpassed by CC1-SSC*mec*-IV in 2023 [35]. CC22 was represented by a single ST type, ST22, in two strains. This follows a reported trend that ST22 has become a major healthcare-associated MRSA strain, replacing ST239, a previously dominant clone of CC22 [52]. The ST22-MRSA-IV reported here has also been reported in recent studies from India and China [34,53]. Singletons of ST1232 formed a separate group containing both MRSA and MSSA and may represent a newly emerging lineage or a highly divergent strain.

## 5. Conclusions

In summary, this study was performed to characterize multidrug-resistant *S. aureus* isolates. All MRSA (27/31) isolates in this study exhibited varying degrees of resistance to non-β-Lactam antibiotics, spanning up to seven different antibiotic classes. Almost all of the MRSA isolates carry the same SCC*mec* and have similar antimicrobial susceptibility patterns. ST764-SCC*mec* II-t045 (14/27, 51.9%) was the main clone identified. This study provides antimicrobial resistance patterns and molecular characteristics of local MRSA in this region of Thailand. This contributes to the existing local epidemiological knowledge of MRSA by highlighting a potential shift in common strains, the identification of a new (untypeable) strain, as well as the potential for international spread of an emerging strain. This highlights the importance of a continued surveillance effort for MRSA as its antimicrobial resistance capability continues to evolve, maintaining its status as one of the major causes of hospital- and community-acquired infections.

## Figures and Tables

**Table 1:** Distribution of antimicrobial resistance profiles observed among reporting MRSA and MSSA.

Number of Resistant Antibiotic Classes	Antibiotic Classes	MSSA	MRSA	PVL Positive
1	β-Lactam (AMP and PEN only)	2	-	-
2	β-Lactam (AMP and PEN only), Folate pathway antagonists	1	-	1
	β-Lactam, Fluoroquinolones	-	1	-
3	β-lactam (AMP and PEN only), Lincosamides, Macrolides	1	-	1
	β-Lactam, Fluoroquinolones, Tetracyclines	-	1	-
	β-Lactam, Lincosamides, Macrolides	-	2	2
4	β-Lactam, Lincosamides, Macrolides, Folate pathway antagonists	-	2	2
	β-Lactam, Fluoroquinolones, Aminoglycosides, Tetracyclines	-	1	-
	β-Lactam, Fluoroquinolones, Lincosamides, Macrolides	-	2	-
5	β-Lactam, Fluoroquinolones, Lincosamides, Macrolides, Tetracyclines	-	2	-
	β-Lactam, Fluoroquinolones, Lincosamides, Macrolides, Aminoglycosides	-	4	-
6	β-Lactam, Fluoroquinolones, Lincosamides, Macrolides, Aminoglycosides, Tetracyclines	-	9	-
	β-Lactam, Fluoroquinolones, Lincosamides, Macrolides, Aminoglycosides, Monoxycarbolic acid	-	1	-
7	β-Lactam, Fluoroquinolones, Lincosamides, Macrolides, Aminoglycosides, Ansamycins, Tetracyclines	-	1	-
	β-Lactam, Fluoroquinolones, Lincosamides, Macrolides, Ansamycins, Tetracyclines, Folate pathway antagonists	-	1	-

MRSA: methicillin-resistant *Staphylococcus aureus*; MSSA: methicillin-susceptible *Staphylococcus aureus*; PVL: Panton–Valentine leucocidin; β-lactams group (AMP—ampicillin, PEN—penicillin, and OXA—oxacillin), Aminoglycosides (GEN—gentamicin), Macrolides (ERY—erythromycin), Lincosamides (CLI—clindamycin), Fluoroquinolones (CIP—ciprofloxacin), Tetracyclines (TET—tetracycline), Ansamycins (RIF—rifampin), Monoxycarbolic acid (MUP—mupirocin), and Folate pathway antagonists (SXT—trimethoprim/sulfamethoxazole).

## Data Availability

The data presented in this study are openly available in BioProject no. PRJNA814829 at https://www.ncbi.nlm.nih.gov/bioproject/?term=PRJNA814829 (accessed on 30 March 2025), reference number 814829.

## Supplementary Materials

The following supporting information can be downloaded at: https://www.mdpi.com/article/10.3390/pathogens14050406/s1, Figure S1: PCR amplification products generated by multiplex PCR assays for SCC*mec* types I–V. Lanes M indicate molecular ladder 100-bp (GeneRuler^TM^); I, II, III, IV, and V indicate SCC*mec* types I–V, respectively.
